# Distribution and Characterization of Quaternary Ammonium Biocides Resistant Bacteria in Different Soils, in South-Western China

**DOI:** 10.3390/microorganisms12081742

**Published:** 2024-08-22

**Authors:** Ziyi Guo, Cunli Qin, Lilan Zhang

**Affiliations:** 1 State Key Laboratory of Coal Mine Disaster Dynamics and Control, Chongqing University, Chongqing 400044, China; zygcqfl@163.com (Z.G.); tsumyee@163.com (C.Q.); 2 Key Laboratory of Three Gorges Reservoir Region’s Eco-Environment, Ministry of Education, Chongqing University, Chongqing 400045, China

**Keywords:** QACs, QAC-resistant bacteria, antibiotic-resistant bacteria, soil, produced water

## Abstract

Quaternary ammonium compounds (QACs) are active ingredients in hundreds of disinfectants for controlling the epidemic of infectious diseases like SARS-CoV-2 (COVID-19), and are also widely used in shale gas exploitation. The occurrence of QAC-resistant bacteria in the environment could enlarge the risk of sterilization failure, which is not fully understood. In this study, QAC-resistant bacteria were enumerated and characterized in 25 soils collected from shale gas exploitation areas. Total counts of QAC-resistant bacteria ranged from 6.81 × 10^3^ to 4.48 × 10^5^ cfu/g, accounting for 1.59% to 29.13% of the total bacteria. In total, 29 strains were further purified and identified as *Lysinibacillus*, *Bacillus*, and *Klebsiella* genus. There, bacteria covering many pathogenic bacteria showed different QACs tolerance with MIC (minimum inhibition concentration) varying from 4 mg/L to 64 mg/L and almost 58.6% of isolates have not previously been found to tolerate QACs. Meanwhile, the QAC-resistant strains in the produced water of shale gas were also identified. Phylogenetic trees showed that the resistant species in soil and produced water are distinctly different. That is the first time the distribution and characterization of QAC-resistant bacteria in the soil environment has been analyzed.

## 1. Introduction

Quaternary ammonium compounds (QACs) are a kind of cationic biocides, consisting of a central ammonium group bonded to alkyl and aromatic substituents [1]. Due to their good property of bactericidal and surface activities, QACs are widely used in various industries and products [2,3,4,5,6]. For example, huge amounts of QACs (concentration > 500 mg/L) are applied in hydraulic fracturing fluid in shale gas exploitation to avoid excessive biofilm formation downhole [7]. For example, it was reported that the sales of global disinfectants had increased by more than 30% compared with that before COVID-19 [8], and more than half of disinfectants used QACs as the active ingredient [9]. Inevitably, their release into the surrounding environment would enlarge the occurrence and dissemination of QAC-resistant bacteria and induce sterilization failure [10]. As counted in 2007 [11], 16 outbreaks of disease caused by QAC disinfection failure were already reported due to the emergence of QAC-resistant bacteria. The occurrence and risk of QAC-resistant bacteria should be paid special attention.

As estimated, about 75% of QACs are discharged into the sewage treatment system, and the rest are released directly into the environment [12]. Furthermore, QACs cannot be completely removed from the treatment system due to the limitations of traditional technology. For example, QAC concentrations in the influent of an Australian municipal sewage plant ranged from 7.7 to 27 μg/L and 1.1 μg/L in the effluent [13]. At the same time, the positive charge and hydrophobicity of QACs make them easy to be adsorbed onto activated sludge and eventually release into the environment with sludge application. Ruan et al. [14] found that their concentrations ranged from 1.12 to 505 mg/kg dry weight in the sludge of 52 municipal wastewater treatment plants in China. Additionally, QAC concentrations in the soil irrigated with the effluent of a sewage treatment plant in Mexico were 81 μg/kg, significantly higher than in other non-irrigated areas [15]. Until now, QACs have been widely found in various environmental media [15,16,17]. Their environmental concentrations are always in gradient and much lower than the action ones, further promoting the emergence of QAC-resistant bacteria.

Many QAC-resistant bacteria have been found in some specific disinfection sites including hospitals, food processing plants, and human settlements. For example, the occurrence frequency of QAC-resistant bacteria in the home environment was reported to range from 20% to 45% [18]. The known resistant bacteria were mainly identified as *Staphylococcus aureus*, *Pseudomonas aeruginosa*, *Klebsiella pneumoniae*, *Salmonella*, and *Listeria monocytogenes* [19,20,21,22,23]. Their resistant mechanisms mainly include biofilm formation, alteration of the cell membrane permeability, overexpression of efflux pump, and acquisition of resistant genes [19,24,25,26]. QACs resistance could also be transmitted between inter- and intra-species because some resistant genes are located in mobile genetic elements (MGEs) ([25]. Furthermore, there is rising concern that QACs could also induce the occurrence of antibiotic resistance via cross-selection or co-selection effect [27,28]. Though QACs have been widely found in various environmental media, the occurrence of QACs resistant bacteria in the environment has not been fully revealed.

Soil is always the final sink of heterogenic pollutants and resistant genes, and QACs could be sequestrated in soil for a long time due to the high affinity of organic matter [27]. Meanwhile, QAC biodegradability could provide a series of concentration gradients, further accelerating the emergence of QAC-resistant bacteria in soil. In particular, an amount of the shared resistance gene was found between soils with clinical settings [29], indicating a high transfer risk from soils to human settlements. Thus, identifying the QAC-resistant bacteria species in the soil environment is helpful for controlling these potential environmental risks.

Thus, 25 soil samples were obtained from a shale gas exploitation area in this study, the QACs concentration was determined, and their QAC-resistant bacteria were screened using 3 mg/L QACs as the selection pressure. Then, the resistant ratio of bacteria was calculated and the species were identified based on the full length of 16S rRNA. The objectives of this study were to (1) determine the occurrence characteristics of QAC-resistant bacteria in different soils and (2) identify their phylogenetic positions and compare the species difference of these soil-resistant bacteria with the specific setting. To the best of our knowledge, this is the first report on the occurrence and distribution characteristics of QAC-resistant bacteria in the soil environment, which is useful for the control of QAC-resistant bacteria spread from surroundings to humans.

## 2. Materials and Methods

### 2.1. Sample Description

**Figure 1:**
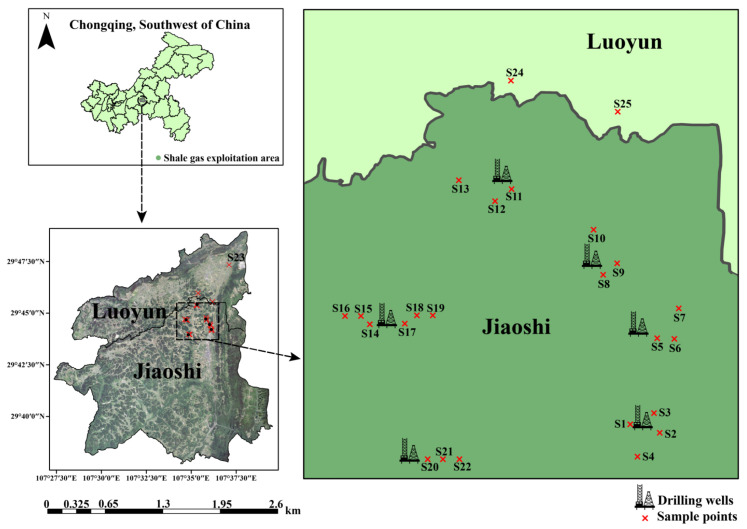
The diagram of soil sample points.

Chongqing, located in Southwest China, has the largest shale gas reservoir in the world except North America. Jiaoshi town is the first shale gas exploitation area in China. It is proposed that the soil has been affected by the exploitation activities. A total of 25 surface (0–20 cm depth) soil samples and 11 produced water samples were collected in this study and numbered in order, respectively. As shown in Figure 1, S1 to S22 were collected from soil around different shale gas drilling wells (exploitation area), while S23 to S25 were collected from soil without drilling wells around (non-exploitation area). These samples covered three soil textures including loam, clay, and sand. The detailed properties of 11 produced water samples can be found in Table S1 in Supplemental Material. All samples were stored at 4 °C until use.

### 2.2. Extraction and Quantification of QACs

The QACs were extracted and quantified using the method reported by the previous study [14,16]. Briefly, approximately 0.2 g of freeze-dried soil was extracted ultrasonically for 1 h with 5 mL hydrochloric acid-methanol (1 M) at 60 °C three times. The supernatant was collected and combined after being centrifuged at 5000 rpm for 5 min. All the supernatant was evaporated to dryness using a vacuum evaporator (RV-10, IKA, German) at 40 °C. The residue was dissolved in 5 × 3 mL ultrapure water and transferred to a 50 mL separatory funnel. Then, the aqueous phase was extracted with 5 mL chloroform three times, and the combined chloroform phase was dried using nitrogen. Finally, the QACs were dissolved in 1.5 mL methanol.

The target QACs (Figure S1) including Alkyltrimethylammonium bromide (ATMAC-10, 12, 14), Benzylalkyldimethylethylammonium chloride (BAC-10, 12, 14), dialkyldimethylammonium bromide (DADMAC-10, 12, 14), recovery surrogate (ATMAC-12D) and internal standard (ATMAC-10D) were quantified by HPLC coupled with triple quadrupole mass spectrometer (LCMS-8060, Shimadzu, Japan). The separation of compounds was conducted using a C18 column (150 × 4.6 mm, 5 μm) at 40 °C. The mobile phases and elution gradients are described in Table S2. A multiple reaction monitoring (MRM) mode was used for detection. The limit of detection (LOD) and limit of quantitation (LOQ) of selected QACs ranged from 0.007 to 0.095 ng/g and 0.022 to 0.317 ng/g, respectively. The concentration of all target QACs was calculated based on the calibration curves for QACs standards. More detailed information is given in Table S3.

### 2.3. Isolation of QAC-Resistant Bacteria

Soil samples were stored at −20 °C and resuscitated for 7 days before enumerations to restore bacterial activity. The action concentration of QACs ranges from 0.5 mg/L to 5 mg/L [30]. Therefore, alkyl (68% C12, 32% C14) dimethyl benzyl ammonium chloride (BAC) (Shanghai Macklin Biochemical Co., Ltd., Shanghai, China) at 3 mg/L was selected for screening QAC-resistant bacteria using LB agar plates according to the previous study [31]. Specifically, 0.5 g dry weight of soil was spiked normal saline, then 50 μL of suspension was coated on sterile LB agar medium containing 3 mg/L BAC and then incubated at 37 °C for 24 h. Then, the colonies on the plates were counted as cfu/g. Colonies enumerations were presented as arithmetic means of triplicate assays. The proportion of QAC-resistant bacteria was determined by dividing the QAC-resistant colonies (cfu/g) by the total colonies (cfu/g) obtained by LB agar without BAC. The screened QAC-resistant bacteria were isolated and purified at least three times via plate streaking, then stored with 25% glycerol at −20 °C. QAC-resistant bacteria in the produced water were also screened and preserved in the same method, but their overabundance caused the failure of colony calculation.

### 2.4. Antibiotic Susceptibility Test

QACs can also enhance bacterial antibiotic resistance due to the similar mechanism of action and target. Thus, 6 antibiotics were selected for antibiotic susceptibility test, including amoxicillin (AMO), vancomycin (VAN), trimethoprim/sulfamethoxazole (T/S), cephalexin (CHA), tetracycline (TET), and Ofloxacin (OFX). The reason for choosing these 6 antibiotics is their huge consumption in China or higher environmental risk [32]. In particular, VAN is the last line of defense against Gram-positive bacterial infection. All antibiotic discs were purchased from Bkman Biotechnology, Hunan, China.

Antibiotic susceptibility test was carried out on QAC-resistant bacteria by using an antibiotic disc, as recommended by the Clinical and Laboratory Standards Institute (CLSI) [33]. Briefly, the culture was diluted to 0.5 McFarland (1.5 × 10^8^ cells), then the diluted culture was dipped with a sterile swab and scribed on the Mueller–Hinton agar medium. The petri dish was rotated 60° and repeated the scribing three times. Then, the edge of the agar was smeared with a swab and stood at room temperature for 3–5 min to dry the agar surface. Finally, the antibiotic discs were stuck on the surface of the plate. The plates were placed at 37 °C for 24 h. The bacteriostatic circle diameter was measured with a vernier caliper for judging the antibiotic susceptibility.

### 2.5. Strains Identification and Minimum Inhibitory Concentration of QACs Determination

The screened resistant bacteria were identified by determining the sequence of 16S rRNA. Firstly, strain DNA was extracted using a Bacterial DNA kit (Omega Bio-Tek, Norcross, GA, USA), then the polymerase chain reaction (PCR) was used to amplify the full-length bacterial 16S rRNA gene using the forward primer 27F (AGRGTTYGATYMTGGCTCAG) and the reverse primer 1492R (RGYTACCTTGTTACGACTT). Then the amplicons were nucleotide sequenced by Sangon Biotech (Shanghai) Co., Ltd., Shanghai, China. The results were compared with the previously saved sequences in the National Center for Biotechnology Information (NCBI, https://www.ncbi.nlm.nih.gov/ accessed on 7 June 2024), and the strain was identified as its best similarity.

The minimum inhibitory concentration (MIC) of QACs for these bacteria was tested according to the Clinical and Laboratory Standards Institute [33]. Briefly, LB medium containing BACs with a concentration ranging from 0 to 512 mg/L (double dilution) were prepared. The overnight cultured bacteria were centrifuged at 5000 rpm for 5 min, discarding the supernatant. Bacteria pellets were resuspended with fresh LB medium and diluted to 0.5 × McFarland (about 10^8^ cfu/mL). Then, 100 μL diluted culture and 100 μL LB medium containing different BAC concentrations were added in 96 well plates, at 37 °C for 24 h. After culture, the MIC for each bacterium was determined as no growth at this concentration.

### 2.6. Statistical Analysis

*T*-test and Pearson correlation analysis were adopted to evaluate the difference of QACs resistant bacteria and the correlation of the proportion of QACs resistant bacteria with physicochemical properties of soil using IBM SPSS Statistics ver. 21.0 (https://www.ibm.com/support/pages/spss-statistics-210-available-download accessed on 7 June 2024). The proportion of QACs resistant was plotted by Origin 2017. The correlations among the proportion of QACs resistant bacteria, physicochemical properties, and heavy metals in soil were obtained by the *R* program language. Nucleic acid sequences were assembled by DNAMAN ver. 9.0. The phylogenetic tree was constructed with MEGA 7.0.

## 3. Results and Discussion

### 3.1. QACs and Heavy Metal Distribution in Soils

**Figure 2:**
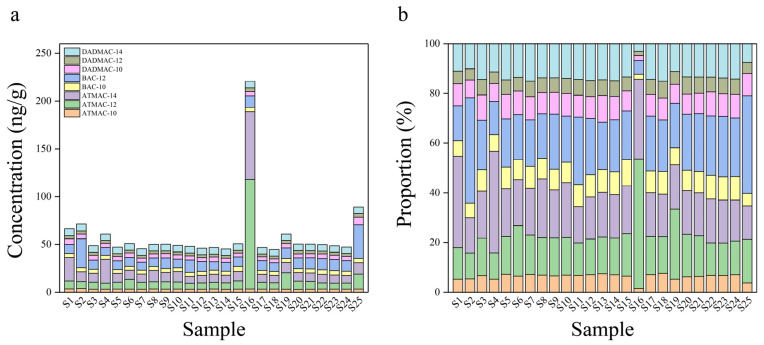
Concentration and composition of QACs in 25 soil samples. (**a**). The concentration of QACs in soil; (**b**). The proportion of different types of QACs in soil.

The detailed soil properties and land utilization type can be found in the supplemental material (Tables S4 and S5). Then, the eight most used QACs were extracted from soil samples with recovery rates ranging from 76.33% to 92.28%, and their concentrations are shown in Figure 2. All target QACs except BAC-14 were detected in all 25 samples, and their total concentration ranged from 44.73 ng/g to 220.50 ng/g with an average concentration of 59.44 ng/g, indicating that the soils were widely polluted by QACs. However, the average concentration is lower than that of the soils irrigated with wastewater at the concentration of 81 ng/g QACs [15]. The highest concentration QACs of 220.50 ng/g were found in the S16 location, which might be due to that its higher organic content elongated the retention of QACs [34]. Meanwhile, the concentration proportion of QACs homologs was positively correlated with their carbon chain length (see Figure 2b); that is, their proportion decreased in the following order: ATMAC-14 (20.11%) > ATMAC-12 (16.84%) > ATMAC-10 (6.29%), BAC-12 (21.46%) > BAC-10 (8.04%), DADMAC-14 (12.93%) > DADMAC-10 (8.62%) > DADMAC-12 (5.71%). This may be due to the greater consumption amount of long-chain QACs for their better bactericidal effect, and the faster biodegradability of the short-chain ones [12,35]. Though BAC and DADMAC were reported to be always added in the shale gas exploitation, there was no significant difference between S1–S22 (exploitation area) and S23–S25 (non-exploitation area), as shown in Table S6.

### 3.2. Proportion of QAC-Resistant Bacteria from Soil

**Figure 3:**
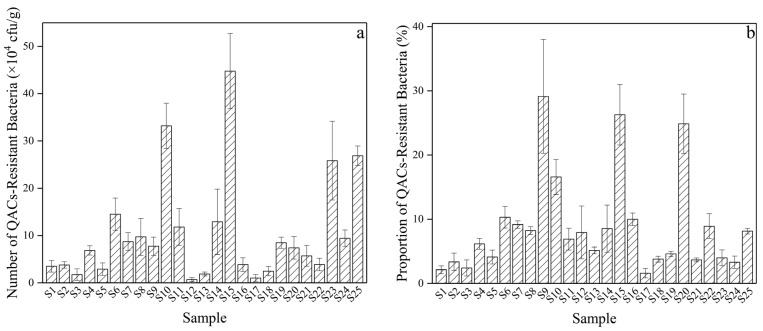
Spatial distribution of number (**a**) and proportion (**b**) of QAC-resistant bacteria of soil samples. (S1 to S22 were around the drilling wells, while S23 to S25 were not adjacent to the drilling wells).

As shown in Figure 3, QAC-resistant bacteria were found in all 25 soil samples with different land use covering all 3 soil types. QAC-resistant bacteria numbers showed significant spatial differences ranging from 6.81 × 10^3^ to 4.48 × 10^5^ cfu/g, and the proportion of resistant bacteria in the 25 soil samples varied from 1.59% to 29.13%, which was relatively high in S9 (29.13%), S15 (26.28%), and S20 (24.85%). *T*-test analysis of QAC-resistant bacteria proportion showed a significant difference between S1 to S22 and S23 to S25. In particular, S9 of the highest resistant bacteria proportion is close to one newly constructed drilling well of shale gas.

**Figure 4:**
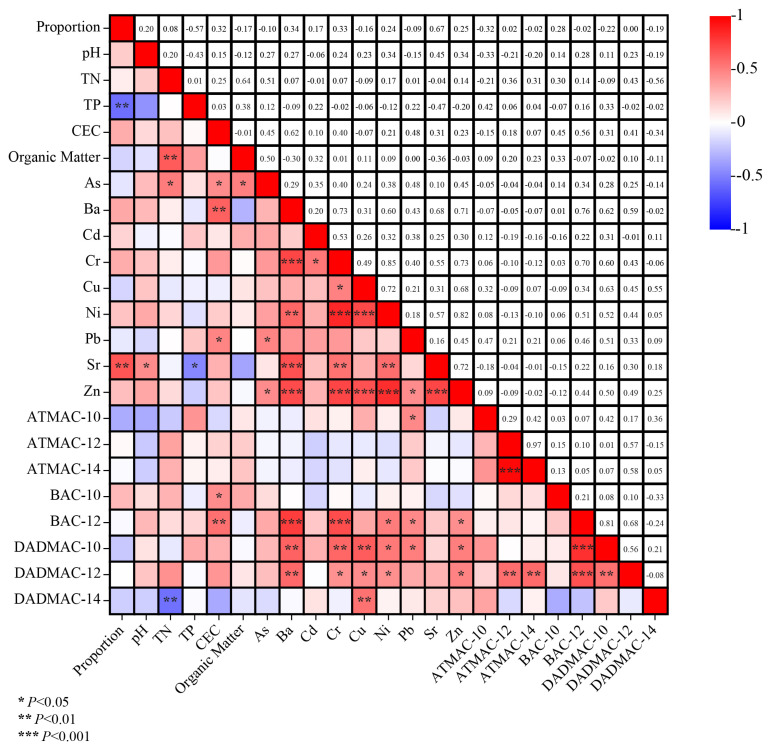
Correlations among the proportion of QACs resistant bacteria, physicochemical properties, and heavy metals in soil. * Correlation is significant at the 0.05 level, ** Correlation is significant at the 0.01 level, *** Correlation is significant at the 0.001 level.

As shown in Figure 4, the correlation among the proportion of QAC-resistant bacteria, soil physicochemical properties (Table S5), heavy metals (Table S7), and QACs concentration was analyzed. The results showed that there was no significant correlation between QAC concentration and the proportion of QAC-resistant bacteria or heavy metals. It might be that QACs could be degraded by soil microorganisms in a few days [36], which might shelter the relationship between the proportion of resistant bacteria and the QAC concentration.

Meanwhile, the proportion of QAC-resistant bacteria was positively correlated with strontium, indicating that strontium might induce the emergence of QAC-resistant bacteria (*p* < 0.01). Furthermore, the highest strontium content of 224.21 mg/kg was found in S9 which had the highest proportion of QAC-resistant bacteria. Heavy metals tend to share resistance mechanisms with QACs, such as efflux pumps and resistance genes, which could be potential co-selects for reducing antimicrobial susceptibility [37,38]. Furthermore, strontium is frequently found in produced water and is always recognized to be brought from the ground with fracturing fluid [39]. Meanwhile, most QACs were also found to have a positive correlation with metal Ba which is always added in hydraulic fracturing fluid in terms of BaSO_4_, suggesting the potential influence of shale gas exploitation activities on the occurrence of resistant bacteria. In addition, the proportion of QAC-resistant bacteria was negatively correlated with the total soil phosphorus.

### 3.3. Antibiotic Resistance Profiles of Isolates

**Figure 5:**
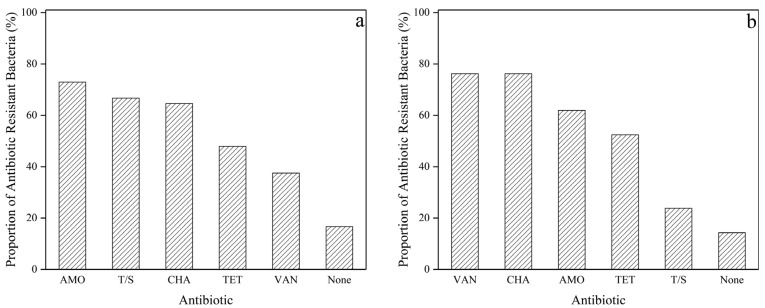
The proportion of antibiotic-resistant bacteria from 69 QAC-resistant bacteria. (**a**). Proportion of antibiotic-resistant bacteria in 48 QAC-resistant bacteria from soil. (**b**). Proportion of antibiotic-resistant bacteria in 21 QAC-resistant bacteria from produced water.

Due to the co-existence of bacteria and QACs, QAC-resistant bacteria should also exist in the produced water of shale gas. Therefore, QAC-resistant bacteria were also screened from the produced water, and a total of 21 QAC-resistant bacteria were selected. A total of 69 QAC-resistant strains were screened from soil and produced water samples. Antibiotics and QACs have similar action mechanisms and target organisms; thus, QAC-resistant bacteria might also be less susceptible to antibiotics. Six antibiotics (AMO, VAN, T/S, CHA, TET, and OFX) were selected for the susceptibility test because of their huge consumption in China [40], and the susceptibility of the 69 strains towards antibiotics are shown in Tables S8 and S9. The proportion of antibiotic-resistant bacteria from QAC-resistant bacteria is shown in Figure 5.

As seen in Figure 5a, the ratio of resistance to antibiotics of 48 QAC-resistant strains from soil was 72.92% for AMO, 66.67% for T/S, 64.58% for CHA, 47.92% for TET, and 37.5% for VAN, respectively, and no strains exhibit OFX resistance. The highest ratio of AMO resistance might be due to its extensive use in personal health care and veterinary medicine [41]. Only 16.67% of QAC-resistant bacteria were not resistant to any of the 6 antibiotics. Moreover, most strains showed multiple resistance (Table S5); for example, 64.58% of the strains showed resistance to both AMO and T/S.

Among the 21 QAC-resistant strains from produced water (Figure 5b), the ratio of resistance was 76.19% for VAN, 76.19% for CHA, 61.90% for AMO, 52.38% for TET, and 23.80% for T/S. Similarly, no strains showed OFX resistance and only 14.29% were not resistant to any of the 6 antibiotics. In contrast, the highest ratio of VAN resistance and CHA resistance was observed in the produced water. VAN is deemed as the last line of defense in treating Gram-positive pathogen infection and the presence of VAN-resistant bacteria in soil and produced water should be paid more attention. The absence of any antibiotics in the produced water suggested that the antibiotic resistance of these bacteria might be caused by the amounts of chemicals in the produced water. For example, Vikram et al. [42] found that bacteria can increase antimicrobial tolerance via osmotic stress regulation/adjustment, changes in membrane permeability, energy synthesis and conversion, and protein transport after produced water exposure.

In summary, most QAC-resistant strains from soil and produced water were also resistant to antibiotics. The mechanism of QAC-resistant bacteria to antibiotics might be due to cross-resistance and co-resistance [43], especially for the bacteria from produced water, which has no chance for their bacteria to be exposed to any antibiotics. Cross-resistance refers to the simultaneous development of QACs and antibiotic resistance by bacteria via biofilm formation and membrane permeability alteration, which have been reported by many researchers. Co-resistance of QAC-resistant bacteria to antibiotics could be attributed to the QAC-resistance gene and antibiotic gene located on the same plasmid. However, it is hard to say whether QACs or antibiotics exactly induced the resistant bacteria due to the co-occurrence of both pollutants, but this could be identified in further lab experiments.

### 3.4. Strains Identification and MIC Determination

As mentioned above, the QAC-resistant and antibiotic-resistant bacteria were widely identified in soil and produced water samples. To further characterize the resistant bacteria in soil and produced water, 44 strains (including four strains without QACs resistance as control) were selected for further identification according to their morphology and resistant types. The sequencing results have been uploaded to the GenBank database, and the full-length 16S rRNA gene was compared to those in the GenBank database to identify the strains with the best similarity, as shown in Table 1. Furthermore, the MIC of selected QACs was further determined and found to range differently from 4 mg/L to 64 mg/L. The bacteria with higher MIC (higher than 16 mg/L) were all from the soils with the shale gas exploitation area.

### 3.5. QAC-Resistant Bacteria in Soil

The 25 resistant bacteria in the soil mainly belonged to *Lysinibacillus, Bacillus*, and *Klebsiella* genus, and almost 58.6% of isolates have not previously been reported to tolerate QACs. Among of them, 17 QAC-resistant strains belonged to *Bacillus*, and 4 strains without QAC resistance were also *Bacillus* genus. This may be due to the wide distribution and strong adaptability of *Bacillus* in the natural environment [44]. *Lysinibacillus*, a ubiquitous Gram-positive bacteria bacterium in the environment, could promote crop growth and kill nematodes and mosquitoes without threatening human health [45,46,47]. No literature is available on their resistance analysis. We proposed that the ability of *Lysinibacillus* to resist QACs may be due to the formation of endospores which could enhance the stress resistance of bacteria [48].

Among the 17 QAC-resistant strains belonging to *Bacillus*, 10 strains were *Bacillus cereus*, 3 strains were *Bacillus* sp., 2 strains were *Bacillus thuringiensis*, 1 strain was *Bacillus licheniformis*, and 1 strain was *Bacillus marisflavi*. *Bacillus cereus* is a Gram-positive pathogen that is well-known for bacterial foodborne disease [49]. They can resist QACs by upregulating the expression of genes involved in fatty acid metabolism [50,51]. These genes participate in the *β*-oxidation process of fatty acid metabolism by regulating the synthesis of acetyl coenzyme A, acetyltransferase, and enoyl-CoA hydratase, and then changing the permeability of cell membrane via increasing the content of short-chain fatty acid [50]. At the same time, 10 strains identified as *Bacillus cereus* (S1-2, S1-3, S2-1, S3-3, S5-1, S9-1, S10-2, S17-1, S23-1, and S24-3) were not only resistant to QACs but also to AMO. Multi-drug-resistant *Bacillus cereus* could undoubtedly threaten human health. On the one hand, QAC resistance could lead to disinfection failure, thus increasing the risk of *Bacillus cereus* transmission through food. On the other hand, since AMO is mainly used to kill Gram-positive bacteria, AMO resistance could increase the difficulty of treatment of diseases caused by *Bacillus cereus*. The resistance mechanism of non-pathogenic bacteria is unclear. Due to their close relationship with pathogenic bacteria, whether the resistance gene transfer will occur between pathogenic and non-pathogenic bacteria deserves further study.

Another large class of resistant bacteria was identified as *Klebsiella pneumoniae* (S1-2, S10-1, S11-3, S13-1, S25-1) and *Klebsiella variicola* (S14-2), both of which were Gram-negative pathogens. They could cause sepsis, pneumonia, and organ or tissue infection [52,53]. The resistance mechanism of *Klebsiella pneumoniae* to QACs is mainly through the regulation of the synthesis of efflux pump. For instance, the ArcAB-TolC efflux pump system consisting of multiple proteins also has a wide range of substrates including QACs [19]. The *qacA*, *qacE*, *qac*△*E,* and *cepA* are present in a plasmid that enables *Klebsiella pneumoniae* to develop resistance to QACs by coding proton pump [19,54,55]. At present, there is no report on whether *Klebsiella variicola* is resistant to QACs, indicating that it is acquired resistance. This is because the QAC resistance genes located in MGEs can be transmitted between homologous bacteria through horizontal gene transfer (HGT) [25], and *Klebsiella variicola* belong to *Klebsiella* genus as well as *Klebsiella pneumoniae*.

### 3.6. QAC-Resistant Bacteria in Produced Water

The QAC-resistant bacteria in produced water were mainly *Exiguobacterium*, *Serratia*, *Brucella*, *Pseudomonas*, *Achromobacter*, *Enterobacter*, *Acinetobacter* genus, and all of them are Gram-negative except *Exiguobacterium*. This could explain the prevalence of VAN resistance in the produced water, as VAN mainly targets Gram-positive pathogens. Among the 11 strains identified, 7 strains were pathogenic, including PW3-2 (*Serratia*), PW4-1 (*Brucella melitensis*), PW5-3 (*Klebsiella pneumoniae*), PW7-2 (*Achromobacter*), PW10-3 (*Klebsiella*), PW10-4 (*Klebsiella pneumoniae*), and PW11-1 (*Acinetobacter*). In particular, PW3-2 (*Serratia*) and PW4-1 (*Brucella melitensis*) are pathogens that seriously endanger human health and are often accompanied by antimicrobial resistance [56,57,58], such as VAN, CHA, and AMO resistance. PW7-2 (*Achromobacter*) is an emerging pathogen that causes chronic respiratory infection in cystic fibrosis patients [59]. PW11-1 (*Acinetobacter*) has become a major public health concern due to its high infection and mortality worldwide [60,61].

PW5-2 was identified as *Pseudomonas balearica,* which can participate in the nitrogen cycling process and improve crop drought resistance [62,63]. *Pseudomonas* was a common genus with QAC resistance and had the ability to biodegrade QACs [12]. *Pseudomonas* mainly reduce intracellular QACs by synthesizing efflux pumps and changing the permeability of cell membranes. It can synthesize MuxABC-OpmB and QacE pump by upregulating the expression of *muxABC-opmB* and *qacE* located in chromosome and plasmid to resist QACs [64,65]. *Pseudomonas* can also decrease the expression of *oprG* coding for OprG, which is one of the major proteins in the outer membrane, to prevent QACs from entering the cell [26,66]. To the best of our knowledge, there is no data on the mechanism of QACs resistance of *Pseudomonas balearica*, which could be the result of the acquisition of resistance through HGT or multiple pressures in produced water.

### 3.7. Phylogenetic Analysis on All the Resistant Bacteria

**Figure 6:**
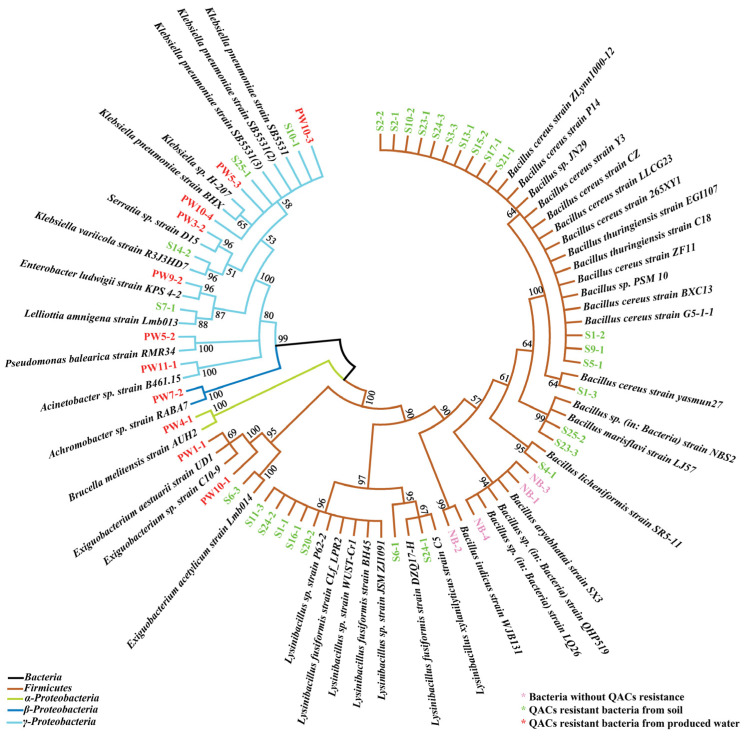
Phylogenetic tree of selected isolates was constructed based on the nucleotide sequences of 16S rRNA. Bootstrap values were only shown for nodes that had >50% support in a bootstrap analysis of 1000 replicates.

A phylogenetic tree was constructed to analyze the correlations between resistant bacteria in soil and produced water, as shown in Figure 6. It could be clearly seen that PW9-2, PW5-2, PW11-1, PW7-2, PW4-1, PW1-1, and PW10-1 were more phylogenetically related, suggesting that there were differences in community structure of resistant bacteria between soil and produced water. The resistant bacteria in the soil environment might be derived from the induction of stressors not the leakage of resistant bacteria from produced water. QAC-resistant bacteria in soil and produced water were classified according to the phylum, and QAC-resistant bacteria in soil belonged to *Firmicutes* and *γ-Proteobacteria*. While QAC-resistant bacteria were relatively abundant, including *Firmicutes*, *α-Proteobacteria*, *β-Proteobacteria,* and *γ-Proteobacteria*. Meanwhile, the QAC-resistant bacteria in produced water have been exposed to extreme environments where multiple pollutants coexist for a long time, indicating that these bacteria are highly adaptable. Once the produced water leaks into the soil environment, it might cause biological invasion due to its competitive advantage, posing a potential risk to ecological safety and human health. Therefore, it is necessary to take more management measures for the disposal of produced water.

## 4. Conclusions

The distribution of QAC-resistant bacteria in the soils of shale gas exploitation areas was studied for the first time using QACs as the selection pressure. The results found that QAC-resistant bacteria were widely present in the soils with the highest resistance ratio of 29.13%. And, shale gas exploitation activities might promote the emergence of QAC-resistant bacteria via the co-selection of unique heavy metal pressure. Furthermore, most soil-resistant bacteria also show co-resistance to antibiotics, showing potential risks to human health and the environment. Strain identification showed that the resistant bacteria in the soil were mainly *Bacillus*, *Lysinibacillus,* and *Klebsiella* genus. More importantly, of the 40 QAC-resistant isolates screened from soil and produced water, 47.5% belong to pathogens. Except for the known 17 resistant bacteria, the resistant mechanisms of the rest 23 species were not revealed, which is not beneficial for controlling the risk of QAC usage.

## Figures and Tables

**Table 1:** Accession number of 16S rRNA and the closest phylogenetic relationship of the isolated bacteria in this study.

Isolates	Accession Number	Species of Closest GenBank Match	MIC (mg/L)	Similarity (%)
NB-1	ON680914	*Bacillus* sp. *(in: Bacteria) strain LQ26* (MG025786)	2	99.86
NB-2	ON680916	*Bacillus indicus strain WJB131* (KU877665)	2	99.51
NB-3	ON680903	*Bacillus* sp. *(in: Bacteria) strain QHP519* (MZ956306)	2	99.45
NB-4	ON680888	*Bacillus aryabhattai strain SX3* (MF431749) (MF431749)	2	99.66
S1-1	ON692704	*Lysinibacillus* sp. *strain P62-2* (MN199553)	4	99.59
S1-2	ON680889	*Bacillus cereus strain ZLynn1000-12* (KY316446)	4	99.24
S1-3	ON680884	*Bacillus cereus strain yasmun27* (OK632089)	4	99.04
S2-1	ON680907	*Bacillus cereus strain P14 16S* (JN700160)	8	99.11
S2-2	ON680894	*Bacillus* sp. *JN29* (KC121050)	8	99.38
S3-3	ON692756	*Bacillus cereus strain Y3* (GQ462534)	8	99.66
S4-1	ON680869	*Bacillus licheniformis strain SR5-11* (MN421512)	4	99.17
S5-1	ON692755	*Bacillus cereus strain CZ* (JX544747)	8	99.1
S6-1	ON680913	*Lysinibacillus xylanilyticus strain C5* (KX832684)	4	99.17
S6-3	ON692705	*Exiguobacterium acetylicum strain Lmb014* (KT986087)	4	99.31
S7-1	ON680915	*Lelliottia amnigena strain Lmb013* (KT986086)	64	98.61
S9-1	ON680883	*Bacillus cereus strain LLCG23* (GU568201)	32	99.38
S10-1	ON692757	*Klebsiella pneumoniae strain SB5531* (MK040621)	8	98.89
S10-2	ON680886	*Bacillus cereus strain 265XY1* (KF818648)	4	99.31
S11-3	ON692762	*Lysinibacillus fusiformis strain CLf_LPR2* (MH788986)	4	99.86
S13-1	ON692764	*Bacillus thuringiensis strain EGI107* (MN704423)	8	99.72
S14-2	ON680898	*Klebsiella variicola strain R3J3HD7* (MK855126)	16	99.31
S15-2	ON692763	*Bacillus thuringiensis strain C18* (KX832697)	8	99.34
S16-1	ON692739	*Lysinibacillus* sp. *strain WUST-Cr1* (KX096881)	4	99.45
S17-1	ON692767	*Bacillus cereus strain ZF11* (KX784916)	8	99.24
S20-2	ON692738	*Lysinibacillus fusiformis strain BH45* (KY910256)	4	99.11
S21-1	ON692766	*Bacillus* sp. *PSM 10* (JF738149)	8	99.72
S23-1	ON680946	*Bacillus cereus strain BXC13* (MN227491)	8	99.11
S23-3	ON692768	*Bacillus marisflavi strain LJ57* (MG049773)	4	98.77
S24-1	ON692770	*Lysinibacillus fusiformis strain DZQ17-H* (HQ143586)	4	98.56
S24-2	ON692769	*Lysinibacillus* sp. *strain JSM ZJ1091* (MW627424)	4	98.23
S24-3	ON680905	*Bacillus cereus strain G5-1-1* (MN595060)	8	99.48
S25-1	ON692774	*Klebsiella pneumoniae strain BHX* (MZ389307)	8	98.41
S25-2	ON692772	*Bacillus* sp. *(in: Bacteria) strain NBS2* (MK757929)	4	99.52
PW1-1	ON692694	*Exiguobacterium aestuarii strain UD1* (MW192903)	8	99.38
PW3-2	ON692744	*Serratia* sp. *strain D15* (MZ342895)	64	98.69
PW4-1	ON680868	*Brucella melitensis strain AUH2* (EF187230)	16	97.89
PW5-2	ON680909	*Pseudomonas balearica strain RMR34* (KT731542)	8	98.82
PW5-3	ON692743	*Klebsiella pneumoniae strain SB5531* (MK040621)	4	98.54
PW7-2	ON680887	*Achromobacter* sp. *strain RABA7* (MN022536)	32	99.3
PW9-2	ON692753	*Enterobacter ludwigii strain KPS 4-2* (JQ308602)	64	99.51
PW10-1	ON692692	*Exiguobacterium* sp. *strain C10-9* (MG757525)	4	98.9
PW10-3	ON692751	*Klebsiella* sp. *H-207* (JX455816)	4	98.96
PW10-4	ON680930	*Klebsiella pneumoniae strain SB5531* (MK040621)	16	97.47
PW11-1	ON680870	*Acinetobacter* sp. *strain B461.15* (OM282819)	16	98.68

MIC means BACs minimum inhibitory concentration for bacteria, NB means the bacteria screened from soil without QAC-resistant, S means the bacteria screened from soil, PW means the bacteria screened from the produced water.

## Data Availability

Data are contained within the article and supplementary materials.

## Supplementary Materials

The following supporting information can be downloaded at: https://www.mdpi.com/article/10.3390/microorganisms12081742/s1, Figure S1: Structural formula of the target QACs; Table S1: Physicochemical properties of produced water samples in different periods; Table S2: The mobile phases and elution gradients procedure; Table S3: The targeted analytes, retention time, monitored multiple reaction monitoring (MRM) transitions; Table S4: Calibration curves for QACs standards; Table S5: Longitude, latitude, and land use types of soil sampling points; Table S6: Physicochemical properties of soil samples; Table S7: The difference of QACs concentration between exploitation area and non-exploitation area; Table S8: Content of heavy metals in soil samples; Table S9: Antibiotic resistance profiles of 48 strains of soil QACs-resistant bacteria; Table S10: Antibiotic resistance profiles of 21 strains of produced water QACs-resistant bacteria.
