# Supplementary material for: Distribution and Characterization of Quaternary Ammonium Biocides Resistant Bacteria in Different Soils, in South-Western China
Source: Microorganisms. 2024 Aug 22;12(8):1742. doi: 10.3390/microorganisms12081742 (PMC11357233; doi:10.3390/microorganisms12081742)
Supplement: Supplementary file 1 [file microorganisms-12-01742-s001.zip › microorganisms-3145982-supplementary.pdf]

# Supplemental material

## Distribution and characterization of quaternary ammonium biocides resistant bacteria in different soils

Ziyi Guo, Cunli Qin, Lilan Zhang*

Key Laboratory of Three Gorges Reservoir Region's Eco-environment, Ministry of Education, Chongqing University, Chongqing, 400045, China

* Corresponding Author: Lilan Zhang

* Key Laboratory of Three Gorges Reservoir Region's Eco-environment, Ministry of Education, Chongqing University, Chongqing, 400045, China.

E-mail: lilanzhang@cqu.edu.cn

**This Supplemental Material Contains the Following Sections:**

Figure S1. Structural formula of the target QACs.

Table S1. Physicochemical properties of produced water samples in different periods.

Table S2. The mobile phases and elution gradients procedure.

Table S3. The targeted analytes, retention time, monitored multiple reaction monitoring (MRM) transitions.

Table S4. Calibration curves for QACs standards.

Table S5. Longitude, latitude, and land use types of soil sampling points.

Table S6. Physicochemical properties of soil samples.

Table S7. The difference of QACs concentration between exploitation area and non-exploitation area.

Table S8. Content of heavy metals in soil samples.

Table S9. Antibiotic resistance profiles of 48 strains of soil QACs-resistant bacteria.

Table S10. Antibiotic resistance profiles of 21 strains of produced water QACs-resistant bacteria.

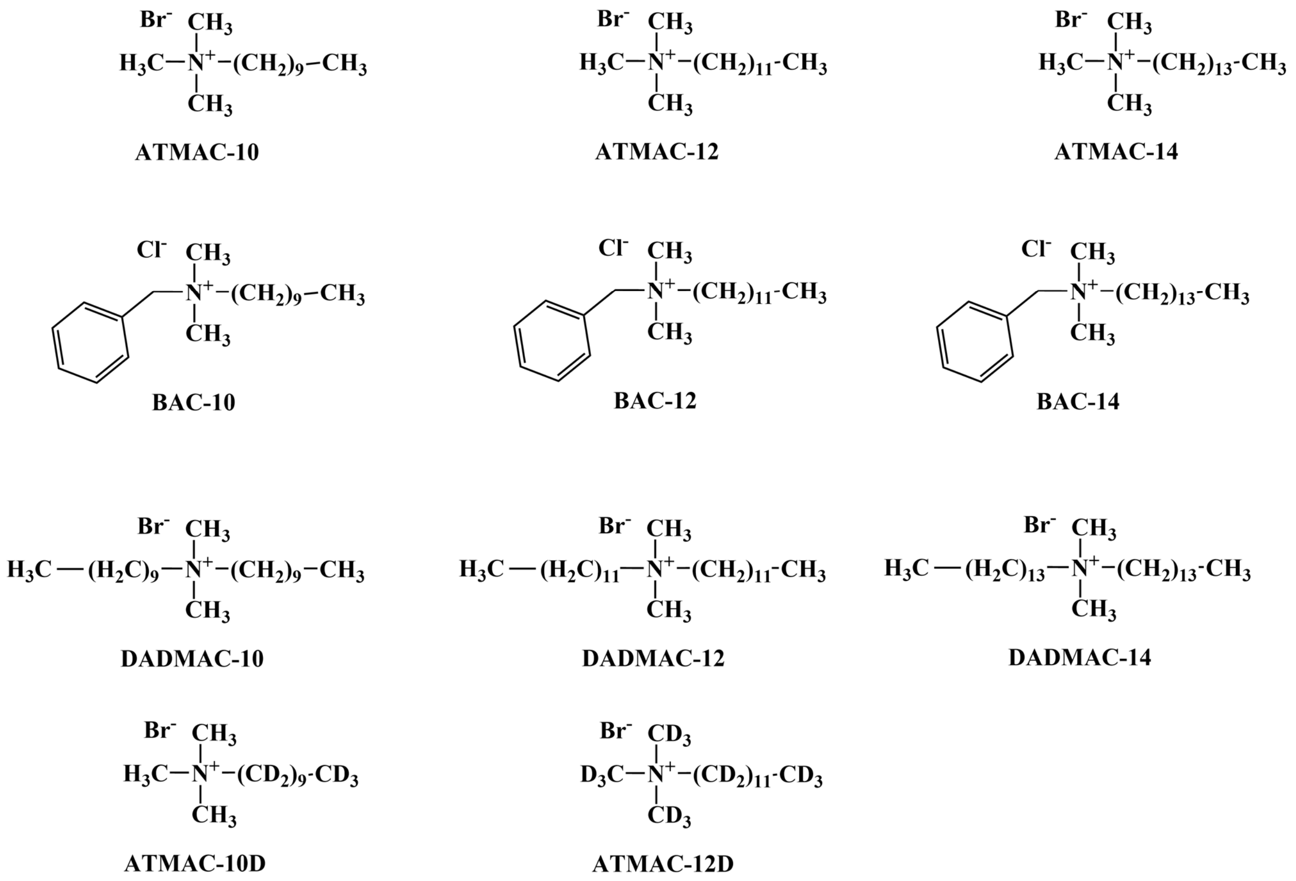


Figure S1. Structural formula of the target QACs.

Table S1. Physicochemical properties of produced water samples in different periods.

| Sample | COD（mg/L） | TOC（mg/L） | BOD5（mg/L） | $Cl^-$（mg/L） | pH |
|---|---|---|---|---|---|
| 1 | 1880 | 620.55 | 949.74 | 5611.8 | 7.8 |
| 2 | 1800 | 601.99 | 760.63 | 4888.8 | 7.94 |
| 3 | 1600 | 455.11 | 678.41 | 4954.5 | 7.31 |
| 4 | 1696 | 512.23 | 711.30 | 5134.5 | 7.57 |
| 5 | 1632 | 461.35 | 703.08 | 5646.5 | 7.65 |
| 6 | 1344 | 342.79 | 548.65 | 5789.1 | 7.62 |
| 7 | 1288 | 304.02 | 603.37 | 5923.5 | 7.36 |
| 8 | 1208 | 280.01 | 512.17 | 5350.7 | 7.42 |
| 9 | 1600 | 231.56 | 648.65 | 5662 | 7.26 |
| 10 | 1304 | 108.65 | 543.37 | 7775.6 | 7.43 |
| 11 | 1192 | 56.38 | 502.17 | 8413.6 | 7.93 |

COD: chemical oxygen demand,

BOD5: 5-days biochemical oxygen demand,

TOC: total organic carbon.

Table S2. The mobile phases and elution gradients procedure

| | Time (min) | Percentage A (%) | Percentage B (%) | Percentage C (%) |
|---|---|---|---|---|
| Elution gradients procedure | 0 | 0 | 80 | 20 |
| | 7 | 80 | 0 | 20 |
| | 13 | 80 | 0 | 20 |
| | 18 | 80 | 0 | 20 |
| | 25 | 0 | 80 | 20 |
| Mobile phases | A | B | C | |
| | Methanol | Pure water | 10 mM $NH_4Ac$ in iso-propanol | |

Table S3. The targeted analytes, retention time, and monitored multiple reaction monitoring (MRM).

| QACs | Retention time (min) | MRM Transitions | MRM transitions for IS |
|---|---|---|---|
| ATMAC-12 | 5.24 | 228.30>60.10 | 228.30>57.20 |
| ATMAC-14 | 7.09 | 256.30>60.10 | 256.30>57.15 |
| BAC-10 | 4.58 | 276.30>91.00 | 276.30>58.15 |
| BAC-12 | 5.75 | 304.30>91.10 | 304.30>58.05 |
| ATMAC-D10 | 4.12 | 221.40>61.10 | 221.40>50.15 |
| ATMAC-D12 | 6.21 | 262.55>105.00 | 262.55>70.20 |
| ATMAC-10 | 4.19 | 200.25>60.20 | 200.25>89.20 |
| BAC-14 | 8.15 | 332.35>91.10 | 332.35>240.25 |
| DADMAC-10 | 9.04 | 326.40>186.20 | 326.40>57.15 |
| DADMAC-12 | 13.70 | 382.45>214.25 | 382.45>57.10 |
| DADMAC-14 | 23.73 | 438.50>242.25 | 438.50>57.15 |

Table S4. Calibration curves for QACs standards

| Compounds | Calibration curves | Linear regression coefficients | LOD (ng/g) | LOQ (ng/g) |
|---|---|---|---|---|
| ATMAC-10 | y = 0.5311x + 0.009 | $R^2 = 0.9958$ | 0.016 | 0.053 |
| ATMAC-12 | y=0.442x+0.0166 | $R^2=0.9963$ | 0.036 | 0.122 |
| ATMAC-14 | y = 0.3645x + 0.0097 | $R^2 = 0.9971$ | 0.095 | 0.317 |
| BAC-10 | y = 0.2707x + 0.0157 | $R^2 = 0.9963$ | 0.024 | 0.081 |
| BAC-12 | y = 0.2158x + 0.0203 | $R^2 = 0.9938$ | 0.072 | 0.239 |
| BAC-14 | y = 0.1654x - 0.0198 | $R^2 = 0.9967$ | 0.012 | 0.039 |
| DADMAC-10:10 | y = 0.3374x + 0.013 | $R^2 = 0.9957$ | 0.007 | 0.022 |
| DADMAC-12:12 | y = 0.2189x + 0.008 | $R^2 = 0.9959$ | 0.010 | 0.033 |
| DADMAC-14:14 | y = 0.2506x + 0.0267 | $R^2 = 0.9934$ | 0.011 | 0.038 |

LOD: limit of detection,

LOQ: limit of quantitation.

Table S5. Longitude, latitude, and land use types of soil sampling points.

| Sampling Point | Longitude | Latitude | Land Use (Crop) |
|---|---|---|---|
| S1 | 107°36′6.85″ | 29°44′13.01″ | Wasteland |
| S2 | 107°36′9.46″ | 29°44′10.44″ | Wasteland |
| S3 | 107°36′7.80″ | 29°44′14.04″ | Wasteland |
| S4 | 107°36′6.28″ | 29°44′9.69″ | Wasteland |
| S5 | 107°36′3.77″ | 29°44′24.84″ | Farmland (Corn) |
| S6 | 107°36′5.40″ | 29°44′24.79″ | Farmland (Corn) |
| S7 | 107°36′2.80″ | 29°44′28.56″ | Farmland (Corn) |
| S8 | 107°35′50.36″ | 29°44′40.22″ | Wasteland (Sichuan Pepper) |
| S9 | 107°35′50.86″ | 29°44′42.53″ | Wasteland |
| S10 | 107°35′49.63″ | 29°44′47.01″ | Wasteland |
| S11 | 107°35′19.22″ | 29°45′22.21″ | Farmland (Corn) |
| S12 | 107°35′17.82″ | 29°45′22.08″ | Wasteland |
| S13 | 107°35′16.55″ | 29°45′23.26″ | Farmland (Corn) |
| S14 | 107°34′40.01″ | 29°44′41.02″ | Farmland (Corn) |
| S15 | 107°34′39.21″ | 29°44′41.80″ | Farmland (Corn) |
| S16 | 107°34′38.86″ | 29°44′41.97″ | Farmland (Corn) |
| S17 | 107°34′45.09″ | 29°44′39.12″ | Wasteland |
| S18 | 107°34′46.07″ | 29°44′39.97″ | Wasteland |
| S19 | 107°34′46.47″ | 29°44′39.92″ | Wasteland |
| S20 | 107°34′51.25″ | 29°43′58.92″ | Wasteland |
| S21 | 107°34′54.20″ | 29°43′58.26″ | Farmland (Corn) |
| S22 | 107°34′55.28″ | 29°43′58.29″ | Farmland (Corn) |
| S23 | 107°37′6.56″ | 29°47′20.07″ | Wasteland |
| S24 | 107°35′23.13″ | 29°45′57.81″ | Farmland (Rice) |
| S25 | 107°36′11.00″ | 29°45′33.17″ | Wasteland |

Table S6. Physicochemical properties of soil samples.

| Sample | pH | TN (g/kg) | TP (g/kg) | CEC (cmol/kg) | Organic Matter (g/kg) | Soil Types[a] |
|---|---|---|---|---|---|---|
| S1 | 7.5 | 1.1423 | 0.5769 | 4.654402736 | 21 | Loam |
| S3 | 4.62 | 1.5113 | 0.6388 | 6.090064387 | 24 | Clay |
| S5 | 4.58 | 1.3531 | 1.1098 | 6.574698193 | 22.4 | Clay |
| S6 | 7.75 | 1.3044 | 0.5797 | 8.004635376 | 16.6 | Clay |
| S7 | 5.97 | 1.6894 | 0.9318 | 5.643528991 | 24.9 | Clay |
| S8 | 6.24 | 1.7351 | 0.7286 | 3.38506065 | 25.2 | Clay |
| S9 | 7.42 | 0.8085 | 0.2668 | 7.252036447 | 9.6 | Clay |
| S10 | 5.08 | 1.9063 | 0.6416 | 6.601178647 | 26.7 | Clay |
| S11 | 4.95 | 1.381 | 0.6162 | 9.559883644 | 15.7 | Clay |
| S12 | 7.79 | 0.665 | 0.3558 | 7.271012979 | 6.5 | Clay |
| S13 | 4.48 | 1.1483 | 1.0182 | 9.045844836 | 14.2 | Clay |
| S14 | 6.94 | 1.9472 | 0.4542 | 4.422590566 | 13.1 | Clay |
| S15 | 6.98 | 1.8677 | 0.4289 | 11.16358276 | 24.3 | Clay |
| S16 | 5.21 | 2.9326 | 0.6912 | 8.661684197 | 27.2 | Loam |
| S18 | 6.71 | 1.7516 | 0.4494 | 6.383603441 | 10.8 | Loam |
| S20 | 8.26 | 3.2523 | 0.3027 | 9.621506807 | 18.1 | Clay |
| S21 | 7.86 | 1.8832 | 0.6665 | 6.020594392 | 24.2 | Clay |
| S22 | 6.34 | 2.2457 | 0.5506 | 4.5987901 | 24.6 | Clay |
| S23 | 7.87 | 3.3473 | 0.7216 | 9.536874008 | 39.6 | Sand |
| S24 | 8.13 | 2.135 | 0.9114 | 7.137998978 | 22.1 | Sand |
| S25 | 7.95 | 1.9493 | 0.8751 | 12.2948131 | 16.9 | Sand |

TN: total nitrogen,

TP: total phosphorus,

CEC: cation exchange capacity,

[a] Classification of soil samples according to “Chinese soil” prepared by Institute of Soil Science, Chinese Academy of Sciences.

Table S7. The difference of QACs concentration between exploitation area and non-exploitation area.

| Compound | ATMAC-10 | ATMAC-12 | ATMAC-14 | ΣATMAC | BAC-10 | BAC-12 |
|---|---|---|---|---|---|---|
| *P-value* | 0.923 | 0.807 | 0.633 | 0.606 | 0.166 | 0.403 |
| Compound | ΣBAC | DADMAC-10 | DADMAC-12 | DADMAC-14 | ΣDADMAC | ΣQACs |
| *P-value* | 0.391 | 0.435 | 0.484 | 0.131 | 0.487 | 0.881 |

Table S8. Content of heavy metals in soil samples.

| Sample | As (mg/kg) | Ba (mg/kg) | Cd (mg/kg) | Cr (mg/kg) | Cu (mg/kg) | Ni (mg/kg) | Pb (mg/kg) | Sr (mg/kg) | Zn (mg/kg) |
|---|---|---|---|---|---|---|---|---|---|
| S1 | 8.79 | 626.22 | 0.27 | 73.98 | 61.69 | 39.32 | 36.33 | 130.18 | 126.53 |
| S3 | 8.43 | 221.17 | 0.28 | 74.67 | 47.44 | 35.26 | 37.87 | 49.65 | 109.44 |
| S5 | 6.32 | 421.48 | 0.24 | 74.94 | 28.89 | 30.75 | 31.13 | 39.30 | 82.11 |
| S6 | 11.13 | 352.80 | 0.34 | 91.77 | 35.41 | 36.89 | 34.27 | 130.08 | 98.45 |
| S7 | 4.46 | 201.54 | 0.35 | 68.35 | 18.69 | 17.78 | 26.81 | 66.48 | 66.94 |
| S8 | 6.67 | 318.45 | 0.32 | 90.59 | 31.51 | 36.75 | 37.43 | 87.48 | 119.39 |
| S9 | 8.22 | 972.78 | 0.39 | 111.01 | 40.54 | 50.97 | 38.18 | 224.21 | 134.29 |
| S10 | 6.55 | 292.13 | 0.58 | 85.85 | 32.36 | 26.06 | 39.14 | 77.71 | 88.78 |
| S11 | 10.35 | 1025.44 | 0.30 | 86.14 | 26.83 | 29.69 | 40.00 | 104.26 | 95.25 |
| S12 | 2.00 | 325.64 | 0.20 | 52.46 | 23.65 | 14.68 | 31.57 | 79.83 | 70.21 |
| S13 | 7.46 | 488.74 | 0.24 | 69.04 | 27.20 | 25.70 | 42.51 | 59.88 | 94.58 |
| S14 | 5.48 | 320.10 | 0.14 | 58.38 | 21.50 | 16.11 | 30.89 | 54.17 | 86.45 |
| S15 | 5.34 | 452.08 | 0.19 | 73.24 | 22.13 | 24.45 | 33.22 | 82.76 | 81.02 |
| S16 | 7.22 | 264.87 | 0.19 | 68.00 | 27.53 | 23.86 | 40.22 | 71.84 | 85.91 |
| S18 | 5.75 | 269.83 | 0.12 | 72.43 | 27.15 | 25.65 | 37.60 | 48.37 | 86.59 |
| S20 | 11.65 | 1553.24 | 0.22 | 90.60 | 33.64 | 47.77 | 29.32 | 220.13 | 138.11 |
| S21 | 3.51 | 47.19 | 0.20 | 81.95 | 41.17 | 37.39 | 23.09 | 47.84 | 86.95 |
| S22 | 4.63 | 111.65 | 0.26 | 81.46 | 36.47 | 44.68 | 26.25 | 36.90 | 82.53 |
| S23 | 38.94 | 305.92 | 0.38 | 84.97 | 34.51 | 37.23 | 41.69 | 53.15 | 111.40 |
| S24 | 7.12 | 260.69 | 0.32 | 64.78 | 28.72 | 27.39 | 38.57 | 87.71 | 92.38 |
| S25 | 18.41 | 2200.63 | 0.39 | 133.55 | 48.23 | 54.60 | 48.00 | 131.09 | 138.78 |

Table S9. Antibiotic resistance profiles of 48 strains of soil QACs-resistant bacteria.

| Antibiotic | Strains | Proportion (%) |
|---|---|---|
| AMO | S1-2, S1-3, S2-1, S2-2, S3-1, S3-2, S3-3, S5-1, S6-2, S7-1, S8-1, S8-2, S9-1, S10-1, S10-2, S11-1, S11-2, S11-3, S13-1, S13-2, S14-1, S14-2, S14-3, S15-1, S15-2, S17-1, S21-1, S23-1, S23-2, S23-3, S24-1, S24-2, S24-3, S25-1, S25-2 | 72.92 |
| T/S | S1-2, S1-3, S2-1, S3-1, S3-2, S3-3, S5-1, S6-1, S6-2, S7-1, S8-1, S8-2, S9-1, S10-2, S11-1, S11-2, S11-3, S13-1, S13-2, S14-1, S14-3, S15-2, S17-1, S21-1, S23-1, S23-2, S23-3, S24-1, S24-2, S24-3, S25-1, S25-2 | 66.67 |
| CHA | S1-2, S1-3, S2-2, S3-1, S3-2, S3-3, S5-1, S7-1, S8-1, S8-2, S9-1, S10-1, S10-2, S11-1, S11-2, S11-3, S13-1, S13-2, S14-1, S14-3 S15-1, S15-2, S17-1, S21-1, S23-2, S23-3, S24-1, S24-2, S24-3, S25-1, S25-2 | 64.58 |
| TET | S1-2, S5-1, S6-1, S6-2, S7-1, S8-2, S10-1, S11-1, S11-2, S11-3, S13-1, S13-2, S14-1, S14-3, S15-1, S21-1, S23-1, S23-2, S23-3, S24-1, S24-2, S25-1, S25-2 | 47.92 |
| VAN | S1-2, S2-1, S2-2, S3-1, S3-2, S3-3, S4-1, S4-2, S4-3, S4-4, S7-1, S8-1, S10-1, S14-2, S14-3, S15-1, S15-2, S17-1 | 37.50 |
| OFX | nd | 0.00 |
| None | S1-1, S2-3, S6-3, S16-1, S18-1, S20-1, S20-2, S21-2 | 16.67 |

nd: not detect

Table S10. Antibiotic resistance profiles of 21 strains of produced water QACs-resistant bacteria.

| Antibiotic | Strains | Proportion (%) |
|---|---|---|
| VAN | PW2-1, PW3-1, PW3-2, PW4-1, PW5-1, PW5-2, PW5-3, PW6-1, PW7-1, PW7-2, PW8-1, PW8-2, PW9-1, PW9-2, PW10-3, PW10-4 | 76.19 |
| CHA | PW2-1, PW3-1, PW3-2, PW4-1, PW5-1, PW5-2, PW5-3, PW6-1, PW7-1, PW8-1, PW8-2, PW9-1, PW9-2, PW10-3, PW10-4, PW11-1, | 76.19 |
| AMO | PW2-1, PW3-1, PW3-2, PW4-1, PW5-1, PW5-3, PW7-1, PW9-1, PW9-2, PW10-2, PW10-3, PW10-4, PW11-1, | 61.90 |
| TET | PW2-1, PW3-1, PW3-2, PW5-3, PW6-1, PW7-1, PW7-2, PW9-1, PW9-2, PW10-3, PW10-4 | 52.38 |
| T/S | PW3-1, PW7-1, PW10-2, PW10-4, PW11-1 | 23.80 |
| OFX | nd | 0.00 |
| None | PW1-1, PW1-2, PW10-1 | 14.29 |

nd: not detect
